# Psychological Outcomes of Multimorbidity: Anxiety, Stress and Depression in Hispanic/Latino Older Adults

**DOI:** 10.1093/geroni/igaf122.059

**Published:** 2025-12-31

**Authors:** Ayse Malatyali, Tom Cidav, Dahee Kim, Janet Lopez, Veronica Decker, Ladda Thiamwong

**Affiliations:** University of Central Florida, Orlando, Florida, United States; University of Central Florida, Orlando, Florida, United States; University of Central Florida, Orlando, Florida, United States; University of Central Florida, Orlando, Florida, United States; University of Central Florida, Orlando, Florida, United States; University of Central Florida, Orlando, Florida, United States

## Abstract

Psychological conditions are significant health concerns among older adults with multimorbidity, particularly in the Hispanic/Latino population, where the prevalence of chronic conditions such as diabetes, hypertension, and obesity are disproportionately high. However, limited evidence exists on how multimorbidity and ethnicity influence different psychological outcomes. This study examines the associations of multimorbidity and ethnicity with anxiety, perceived stress, and depressive symptoms. Using logistic regression and generalized linear models, we analyzed cross-sectional data from 2,730 participants (age ≥ 65) in the Health and Retirement Study (2020). Multimorbidity was assessed based on the presence of up to seven chronic conditions. Psychological outcomes were measured using the 5-Item Beck Anxiety Inventory, Perceived Stress Scale, and the Center for Epidemiological Studies Depression Scale. The mean age was 75.24 years. Women had significantly higher rates of anxiety, stress, and depressive symptoms than men. Hispanic/Latino older adults had 98% higher odds of elevated anxiety and 0.18-point higher depression scores than their peers, while their perceived stress scores were not significantly different. Higher multimorbidity levels were strongly associated with the psychological burden, as individuals with three or more chronic conditions had 2.72 times higher odds of anxiety, 0.79-point higher perceived stress, and 0.62-point higher depressive symptoms compared to those without chronic conditions. Functional difficulty and lower income quartiles (Q1 and Q2) were also significant predictors of poorer psychological health. These findings highlight the disproportionate burden of psychological conditions among Hispanic/Latino older adults with multimorbidity, emphasizing the need for targeted interventions to improve psychosocial well-being in aging populations.

